# Advances in Augmented Reality in Sports Surgery: A Systematic Review

**DOI:** 10.1155/aort/6707884

**Published:** 2025-09-10

**Authors:** Negarsadat Namazi, Yashar Khani, Amirhossein Salmannezhad, Mohammad Behdadfard, Ehsan Safaee, Sanam Mohammadzadeh, Mohammad Nouroozi, Amir Mehrvar

**Affiliations:** ^1^Student Research Committee, Shahid Beheshti University of Medical Sciences, Tehran, Iran; ^2^Student Research Committee, Qazvin University of Medical Sciences, Qazvin, Iran; ^3^Student Research Committee, Birjand University of Medical Sciences, Birjand, Iran; ^4^Student Research Committee, Faculty of Medicine, Shahed University, Tehran, Iran; ^5^Research Center for Evidence-Based Medicine, Iranian EBM Centre: A JBI Centre of Excellence, Tabriz University of Medical Sciences, Tabriz, Iran; ^6^Clinical Research Development Units, Taleghani Hospital, Shahid Beheshti University of Medical Science, Tehran, Iran

**Keywords:** arthroscopy, augmented reality, orthopedic surgery, sports surgery

## Abstract

**Purpose:** Augmented reality (AR) blends computer-generated information with the real environment to support surgical visualization, guidance, and training. In sports surgery, where arthroscopic views constrain depth perception and hand–eye coordination, AR may enhance intraoperative accuracy and efficiency and enable engaging rehabilitation. Novelty: To our knowledge, this is the first systematic review focused specifically on AR across the sports surgery continuum (operative and rehabilitative), synthesizing visualization modalities, use cases, and measured outcomes to identify translational gaps.

**Methods:** We searched MEDLINE (PubMed), Embase, Scopus, and Web of Science (January 2024), registered the protocol on PROSPERO (CRD42024543974), and reported according to PRISMA 2020. Eligibility included preclinical and clinical studies using AR/MR in sports–orthopedic contexts. Risk of bias was assessed using RoB 2 (RCTs), ROBINS-I (nonrandomized studies), and NIH tools for other designs. Qualitative synthesis was structured by AR type, surgical indication/use case, and outcome domain.

**Results and Findings:** Twenty-one articles met the criteria. Sixteen assessed intraoperative applications and four rehabilitation, with knee arthroscopy being the most common. Comparative human studies reported more accurate femoral tunnel placement in ACL reconstruction and shorter operative time in selected workflows, while several studies showed feasibility in simulators/cadavers. Video see-through (VST) and optical see-through (OST) (e.g., HoloLens) were most frequently used.

**Conclusions:** AR shows early promise for guidance, training/telementoring, and postoperative rehabilitation in sports surgery, but current evidence is heterogeneous and often feasibility-focused. Larger, controlled clinical trials with standardized outcome definitions and reporting are needed to confirm benefits, evaluate learning curves and ergonomics, and support integration into operating room workflows.

## 1. Introduction

The augmented reality (AR) overlays computer-generated perceptual information onto a real-world environment, allowing users to remain engaged with their surroundings. There are three main types of AR: (1) projection-based (PB), (2) video see-through (VST), and (3) optical see-through (OST). PB uses projectors to overlay digital content onto physical objects, eliminating the need for headsets. VST integrates complex medical data with live surgical views, revolutionizing how surgeons perceive anatomical structures. OST projects digital images onto transparent lenses within head-up displays, allowing simultaneous viewing of the physical environment and relevant digital information during procedures [1–3].

AR in surgery has demonstrated promising results in laparoscopy (e.g., suturing tasks) [4], neurosurgery (e.g., parasagittal meningiomas) [5], and cardiothoracic surgery (e.g., ventriculoperitoneal shunt operation) [6], offering enhanced preoperative planning, improved spatial understanding of the anatomy, and increased intraoperative safety [7]. Orthopedics and trauma are unsurprising, as these surgical procedures often rely on visual data obtained before and during the operation. Minimally invasive orthopedic surgeries such as arthroscopies are frequently used in sports-related injuries. However, this technique is inherently more challenging to conduct because of the inability to visualize and maneuver freely. AR technologies have the potential to dramatically help train surgeons and navigate faster and with more precision due to better hand–eye synchronization and more immersive visualization. In addition, AR is an excellent tool for preoperative planning and postoperative rehabilitation [8–10].

In this systematic review, we delve into the existing body of literature on AR applications within sports surgery, aiming to consolidate evidence, identify trends, and address gaps. Our primary objective is to elucidate the impact of AR on surgical performance, patient recovery, and overall surgical practice. By synthesizing findings from various studies, we hope to provide valuable insights for clinicians, researchers, and technology developers, ultimately advancing the integration of AR into the sports surgery domain.

## 2. Methods

### 2.1. Protocol and Registration

This review followed the PRISMA 2020 reporting guideline, selected for its emphasis on transparent eligibility, reproducible search strategies, and explicit synthesis methods appropriate to complex, heterogeneous evidence bodies typical of emerging surgical technologies. The protocol was registered in the International Prospective Register of Systematic Reviews (PROSPERO) (CRD42024543974) prior to screening. It is registered in the PROSPERO (registration number: CRD42024543974).

### 2.2. Search Strategy

A comprehensive systematic search was conducted on Medline (via PubMed), Web of Science, Scopus, and Embase databases in January 2024. The search strategy consisted of two main concepts: (1) AR with the following key terms: AR, mixed reality, and extended reality and (2) sports–orthopedic surgeries comprising of the following keywords: anterior cruciate ligament^∗^, ACL, meniscus, rotator cuff, bicep^∗^, tricep^∗^, clavicle, hip, foot, ankle, tendinopathy^∗^, Achilles, shoulder, femoroacetabular, patella, athlete^∗^, osteochondritis, sport, and arthroscopy. Keywords (along with their related MeSH terms in PubMed) were combined using the Boolean operators. Full, database-specific search strings (including Boolean operators, field tags, and filters) are provided in Supporting Table S1.

### 2.3. Eligibility Criteria

Preclinical and clinical research studies using AR technology in sports–orthopedic settings were included. Included study types were RCTs, cohorts, cross-sectional studies, and case series. Studies have to be written in English. Reviews, protocols, conference abstracts, book chapters, and letters were excluded.

### 2.4. Study Selection

We imported the included studies from the databases to Rayyan (an online tool to organize and monitor the screening step of systematic reviews) [11]. Three investigators (N.N., M.B., and S.M.) performed the title/abstract and then full-text screening separately. A full discussion resolved any disagreements in the study selection process, and the third reviewer (Y.K.) was consulted if a consensus could not be reached. We did not compute interrater agreement statistics; this is noted as a limitation.

### 2.5. Data Extraction

Four reviewers (N.N., A.S., M.B., and S.M.) independently extracted the relevant data from all studies that met the inclusion criteria. The data extraction was conducted in Microsoft Excel 2023. We have comprehensively reviewed the studies and then performed the data extraction, compiling a list of relevant characteristics and factors under the following topics: title, first author, year, country, study design, compared groups, AR hardware, brand name, use case, assessed action, surgical procedure, outcome measures, and overall results. Any conflicts in the data extraction process were resolved through discussions between the three investigators, and the fourth reviewer (YK) was consulted if necessary.

### 2.6. Quality Assessment

Two authors (AS and SM) performed the quality appraisal for RCTs through the RoB 2 tool [12]. Regarding the non-RCT studies, we utilized the ROBINS-I tool for assessing the bias [13]. A reciprocal consultation was conducted to settle any disagreements. For other study types, we used the NIH quality assessment tool [14].

### 2.7. Data Synthesis

Given methodological and clinical heterogeneity (designs, models, surgical indications, hardware, and outcome measures), we conducted a structured narrative synthesis. Studies were grouped by (i) AR visualization type (projection-based, VST, and OST), (ii) surgical indication/use case (e.g., portal placement, tunnel targeting, navigation, telementoring, and rehabilitation), and (iii) outcome domains (e.g., accuracy/targeting error, time/efficiency, task success, and user-reported usability/comfort). For comparative studies, we summarize effect direction and magnitude when available. Meta-analysis was not attempted due to incompatible measures and small, feasibility-focused samples.

## 3. Results

**Figure 1:**
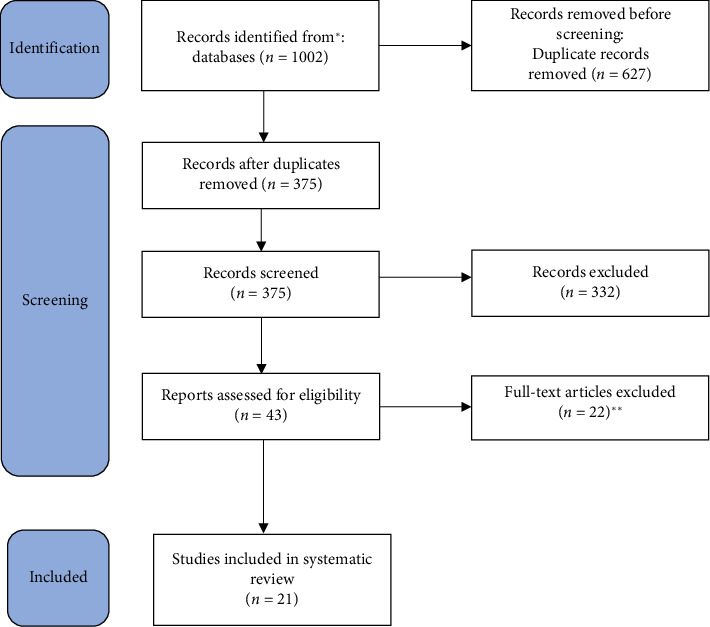
PRISMA flowchart. ^∗^PubMed: 211, Scopus: 409, Embase: 143, and Web of Science: 633. ^∗∗^Not sport surgery related: 9, not AR related: 13.

We conducted a systematic search across 4 databases, including MEDLINE via PubMed, Web of Science, Scopus, and Embase. Our initial search yielded 1002 related articles. After removing 627 duplicates, 375 studies remained for title and abstract screening; of these, 43 were selected for full-text review, and twenty-one articles met our eligibility criteria. Detailed information can be found in the PRISMA flowchart in Figure 1.

Our review includes eight comparative studies that mainly evaluate surgeons' performance. Among the included studies, 16 assessed the AR intraoperatively [10, 15–29], while four focused on AR for rehabilitation [30–33], and one incorporated AR in both preoperative and operative settings [34]. VST visualization with ten studies is the most frequently used. Eight studies used OST for visualization, and five incorporated Microsoft's HoloLens. Further details can be found in Table 1. The outcomes and results of the included studies are summarized in Table 2.

**Figure 2:**
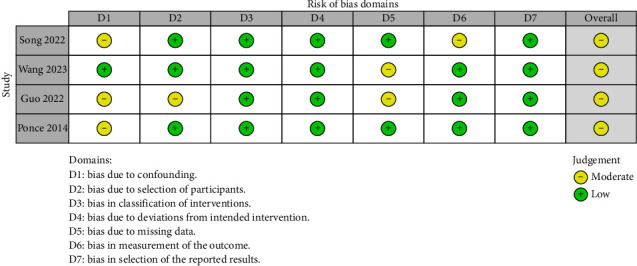
Quality assessment using ROBINS-I for nonrandomized interventional studies.

According to the RoB 2 results, both studies presented “Some concerns” in overall bias judgment [18, 32]. For the domain “Deviations from the Intended Interventions,” both studies presented “Some concerns” and had a “Low risk of bias” for the rest of the domains. We provided more details of our quality assessment in Table 3. Regarding the ROBINS-I analysis, we noticed that all non-RCT interventional studies had a “Moderate” overall risk of bias [19, 21, 23, 26]. Results of the ROBINS-I analysis are illustrated in a traffic light plot in Figure 2. The results of the quality assessment of other studies assessed by NIH quality assessment tools are summarized in Table 4.

## 4. Discussion

Across twenty-one studies, AR was the most frequently applied to intraoperative guidance (portal/tunnel targeting, navigation, telementoring), with additional use in postoperative rehabilitation. VST systems (e.g., monitor-based overlays) and OST head-mounted displays (e.g., HoloLens) predominated, with PB systems used less often. These modalities entail distinct trade-offs in immersion, field of view, sterility, team awareness, and ergonomics that influence deployment decisions in sports surgery [35–38]. The evidence base is early-stage: two RCTs, several small nonrandomized clinical studies, and multiple preclinical/simulator investigations. While comparative results suggest potential improvements in tunnel accuracy and operative time in selected workflows, typical limitations include small samples, short follow-up, and heterogeneity of tasks, hardware, and outcome definitions.

VST integrates digital content with a live video feed of the real world, viewed on a screen or through a headset. This technology significantly enhances surgical precision by combining real-time video with preoperative imaging data, making it particularly useful for minimally invasive surgeries. It is also flexible, as it can be implemented on various devices, including headsets and standard monitors. However, surgeons may initially experience disorientation due to the indirect view of the surgical field, and the quality of the augmented experience depends heavily on the camera and display technology. Examples of VST AR devices include Apple Vision Pro and Meta Quest 3 [39].

OST AR projects digital images onto transparent lenses of head-up displays, such as Google Glass or Microsoft HoloLens. This allows surgeons to view both the physical environment and digital information simultaneously, enhancing situational awareness and precision. OST AR offers an immersive experience and supports hands-free operation through voice commands and gesture recognition, which is crucial for maintaining sterility in surgical environments. However, it may have a limited field of view and can be uncomfortable for prolonged use [40]. By understanding the distinct advantages and limitations of each AR type, we can better appreciate their applications and potential impact on fields such as medicine, where precision and situational awareness are paramount.

Findings align with other specialties, where AR supports preoperative planning, intraoperative targeting, and education/mentoring, yet faces similar barriers (ergonomics, cost, integration). Sports surgery's arthroscopic constraints may particularly benefit from task-specific overlays (e.g., portal vectors and volume frustums) and collaborative telementoring.

To have a broader understanding of advances in these technologies, we explore some noteworthy studies in other fields of surgery. In the context of spine surgery, an extensive review by Ghaednia et al. explores the applications and potential of AR and virtual reality (VR) systems. One of the challenges they highlight is the integration into existing workflows [41]. In cardiothoracic surgery, these applications have guided open, robotic, and endovascular surgery, improving procedural times, reducing dissection, and decreasing patient risk, radiation, and contrast exposure [6, 42, 43]. Pérez et al. published their experience in hepatobiliary–pancreatic surgery. Their study demonstrated the potential for remote holographic connection between surgeons, using virtual consultation models and surgical guides and replacing physical elements with virtual ones [44]. Gouveiaa et al. focus on the future benefits of AR in breast surgery education. They describe two potential applications: impalpable breast cancer localization and surgical remote telementoring, and the technical needs to make it possible [45].

While AR has shown promising advances in sports surgery, considerable challenges may hinder its widespread adoption. For instance, for ergonomic difficulties, such as prolonged use of head-mounted devices causing neck discomfort, Fang et al. [18] noted that the surgeons with head-mounted AR initially had longer completion times than traditional monitors. This is attributed to the ergonomic and usability challenges that decrease with user familiarity [46]. Similarly, training barriers, mainly the steep learning curve associated with integrating AR systems into surgical workflows, were highlighted in several studies [22, 29, 47]. In addition, the high cost of AR technology, as discussed in [5], limits accessibility and adoption, especially in resource-constrained settings. Addressing these challenges will require a collaborative effort among developers, healthcare providers, and policymakers to make AR systems more practical and affordable.

Apple's recently launched Vision Pro headset represents a significant milestone in mixed reality technology for medical applications. While AR and VR have been utilized in medical procedures for years, the Vision Pro introduces groundbreaking processing capabilities and ergonomic improvements. Notably, medical fields such as ophthalmology, neurosurgery, and plastic surgery have already begun integrating this technology. Moreover, the widespread adoption of Apple products provides an advantageous platform for software developers to drive further innovation [48–51]. In addition, AR contact lenses, which directly project content onto the retina and adjust based on eye gaze, hold promise, particularly for enhancing contrast in specific eye diseases [52].

## 5. Conclusion

AR is a promising adjunct for arthroscopic guidance, training/telementoring, and postoperative rehabilitation in sports surgery. Early comparative data indicate potential gains in targeting accuracy and procedure efficiency for selected tasks, while simulator and cadaveric studies support feasibility and skill acquisition. However, the evidence remains heterogeneous, often small-scale, and at risk of bias. Pragmatic, multicenter trials with standardized outcome definitions, ergonomic/learning-curve assessment, and workflow-aware deployment are now the priority to move from feasibility to reliable clinical benefit.

## 6. Limitation

Few articles were published in this field. Most were feasibility studies, not comparative studies. The heterogeneity of the included studies prevented a meta-analysis of their results. The included articles were highly variable regarding study type, surgical procedures, assessed action, and visualization hardware. We did not calculate interrater reliability (*κ*) during screening; disagreements were resolved by consensus with third-reviewer adjudication.

## 7. Future Research Directions

Future research directions should focus on establishing standardized outcome measures for accuracy, efficiency, usability, and safety, supported by adequately powered multicenter trials across common sports procedures. Further work is also needed to quantify learning curves and ergonomics, optimize workflow integration and team interaction, and evaluate cost-effectiveness and equitable access. Finally, studies should address interoperability with surgical and imaging systems while ensuring privacy and security for telementoring applications.

## Figures and Tables

**Table 1:** Study characteristics.

First author/year/country	AR type	Hardware	Use case	Assessed action	Procedure	Model
Bautista/2018/Colombia [15]	OST	META glasses	Operative	Hand gesture detection	Go through menus	Not reported
Chen/2021/China [10]	OST	na	Operative	Landmark targeting	Knee arthroscopy	Knee prosthesis and swine knee
Dario/2000/Italy [17]	na	na	Operative	Landmark targeting	Knee arthroscopy	Knee prosthesis
Fang/2023/Hong Kong [18]	OST	GLOW plus	Operative	Loose-body retrieval	Knee arthroscopy	Knee prosthesis
Fotouhi/2023/USA [20]	OST	HoloLens	Operative	Hand–eye calibration	Spine and pelvic fracture	Pelvis and a spine prosthesis
Franzò/2023/Italy [33]	OST	HoloLens	Rehabilitation	Posture study	Movement restoration	Patient
Garcia/2014/Australia [30]	VST	Mobile phone	Rehabilitation	Movement detection	Ankle rehabilitation	Patient
Guo/2019/China [34]	OST	HoloLens	Preoperative and operative	Landmark targeting	ACL reconstruction	Knee prosthesis
Guo/2022/China [19]	na	na	Operative	Landmark targeting	Scapula fracture	Patient
Jeung/2023/South Korea [16]	VST	Screen	Operative	Landmark targeting	Wrist arthroscopy	Patient and prosthesis
Korn/2019/Germany [31]	PB	na	Rehabilitation	Rehabilitation exercises	Rehabilitation exercise	Patient
Moody/2008/UK [22]	VST	na	Operative	Arthroscopy skills	Knee arthroscopy	Knee prosthesis
Ponce/2014/USA [23]	VST	na	Operative	Hand gesture detection	Shoulder arthroscopy	Patient
Rasool/2013/Singapore [24]	VST	na	Operative	Landmark targeting	Knee arthroscopy	Cadavers
Rose/2015/USA [29]	VST	Screen	Operative	Arthroscopic skills	Arthroscopic training	Virtual reality
Shim/2023/Korea [32]	VST	Screen	Rehabilitation	Rehabilitation exercises	After rotator cuff repair	Patient
Song/2022/Germany [26]	OST	HoloLens	Operative	Landmark targeting	During hip arthroscopy	Hip prosthesis
Stetson/2022/USA [25]	VST	Screen	Operative	Telesurgery mentoring	Arthroscopy	Patient
Tonet/2000/Italy [27]	VST	Screen	Operative	Landmark targeting	Knee arthroscopy	Knee prosthesis
Wang/2023/China [21]	OST	HoloLens	Operative	Bone tunnel positioning	ACL reconstruction	Patient
Zemirline/2013/France [28]	VST	Screen	Operative	Landmark targeting	Wrist arthroscopy	Cadaver

Abbreviations: ACL, anterior cruciate ligament; na, not applicable; OST, optical see-through; PB, projection-based; VST, video see-through.

**Table 2:** Summary of results.

First author/year/country	Summary of technical and clinical results
Bautista/2018/Colombia [15]	In ACL reconstruction surgery, AR interface assists in precise tunnel drilling for graft passage. Evaluating hand-gesture interaction, Bautista et al. compared META glasses (hand recognition) and MYO arm-band (EMG muscle sensors). Results favored the MYO arm-band for efficiency, efficacy, and surgeon satisfaction
Chen/2021/China [10]	In minimally invasive knee surgery, the AR system improves accuracy by 2.70 and 2.10 mm in swine knee and knee phantom, respectively, providing real-time guidance and enhancing anatomical visualization. Virtual arthroscopic images reduce targeting errors significantly
Dario/2000/Italy [17]	In this study, researchers introduce a mechatronic arthroscope, a smart tool for both traditional and computer-assisted arthroscopy. The system combines image processing, trajectory tracking, and an intuitive human–machine interface, enabling virtual navigation and AR guidance. It enhances surgeon control and real-time trajectories for improved intervention accuracy
Fang/2023/Hong Kong [18]	In simulated knee arthroscopy, a prototype wireless AR head-mounted display (AR-HMD) was evaluated against traditional TV monitors (TVMs). Initially, AR-HMD had longer completion times, but with familiarity, it approached TVM performance
Fotouhi/2023/USA [20]	Co-registered head-mounted displays achieved accurate K-wire placement and precise angle measurements in total hip arthroplasty. This holistic approach improves surgical interventions and documentation
Franzò/2023/Italy [33]	A hybrid system that combines virtual and augmented reality technologies, using Microsoft Kinect Azure for motion tracking and Microsoft HoloLens 2 for visualization. They overlaid 3D virtual bone models onto a subject performing karate movements. This system showed promise for monitoring and rehabilitating martial arts athletes after injuries
Garcia/2014/Australia [30]	Addressing the challenge of patient engagement in rehabilitation, this study introduces the Mobile RehApp, an AR application for mobile devices that aims to support ankle sprain rehabilitation by offering interactive exercises. Unlike existing tools, it combines therapeutic benefits with engaging gameplay
Guo/2019/China [34]	An intensity-based 2D–3D registration method for ACL reconstruction navigation. The combination of splatting, Spearman's rank correlation coefficient (SRC), and gradient descent (GD) yielded the best performance. The AR ACL reconstruction navigation system achieved clinical accuracy
Guo/2022/China [19]	They evaluated the feasibility and effectiveness of an AR for treating scapular fractures. Using preoperative virtual simulation and intraoperative navigation, this approach significantly reduced operation time (−28.75 min) and blood loss (−81.94 mL) compared to conventional surgery. The method provides precise planning and safer reduction, with the added benefit of affordability due to the use of a portable projector
Jeung/2023/South Korea [16]	An AR-based surgical guidance system for wrist arthroscopy. By compensating for bone shifts during surgery using noninvasive fiducial markers, they achieved accurate visualization of concealed wrist bones. The method significantly reduced target point errors compared to preoperative CT
Korn/2019/Germany [31]	A system that combines AR and gamification to support elderly rehabilitation. Worn at the waist, the system collects movement data while projecting AR elements onto the user's path. These elements provide location-based information or encourage movement games. Therapists can monitor the data, allowing adaptive challenge levels
Moody/2008/UK [22]	A mixed reality environment using tactile augmentation named the Sheffield Knee Arthroscopy Training System (SKATS). The study explores the effectiveness of mixed reality for knee arthroscopy training
Ponce/2014/USA [23]	A virtual interactive presence (VIP) system was evaluated in an operating room setting. Surgeons found it useful for highlighting anatomy and providing feedback to residents. The system was easy to use, safe, and did not interfere with procedures. Importantly, it enhanced training without increasing operative times
Rasool/2013/Singapore [24]	A novel technique that combines real arthroscopic images with 3D object models. By augmenting the surgical field, this approach allows for photorealistic visualization and haptic interaction. Surgeons can feel the joint cavity as real 3D objects during simulated procedures
Rose/2015/USA [29]	In AR modules for arthroscopy training, experts and intermediate participants outperformed novices in tasks related to centering, triangulation, and coordinated motions. The modules showed construct validity overall and greater arthroscopic experience correlated with improved ambidextrous performance
Shim/2023/Korea [32]	Comparing postoperative rehabilitation methods for patients after rotator cuff repair (RCR), an AR-based system that outperformed conventional rehabilitation. The AR group showed significantly greater improvement in shoulder function, as measured by the simple shoulder test (SST) score, compared to the conventional group. Other outcomes, such as pain, range of motion, and muscle strength, did not differ significantly between the groups
Song/2022/Germany [26]	In hip arthroscopy; an AR system that enhances endoscope placement by overlaying a virtual frustum, indicating the reachable working volume. This approach improves spatial perception and may lead to faster alignment, reduced errors, and improved surgical outcomes
Stetson/2022/USA [25]	Telesurgery platforms create a global network, allowing real-time assistance and collaboration. This study focuses on using the SurgTime telesurgery platform (AR-based technology) to teach arthroscopic surgery skills worldwide.
Tonet/2000/Italy [27]	A prototype AR-based navigation arthroscopy system that features an interactive graphical interface, displaying 3D joint models and real-time tracking of instruments. Positive evaluations by orthopedic surgeons demonstrate its potential
Wang/2023/China [21]	The study compared MR-assisted ACL reconstruction to conventional arthroscopic methods. Results showed that MR improved tunnel positioning (distance from the tunnel's center to the apex of the lateral femoral condyle: 14.07 ± 4.12 vs. 17.49 ± 6.24 mm), with closer alignment to the ideal preoperative design. Both groups of patients experienced significant postoperative improvement based on outcome scores
Zemirline/2013/France [28]	This study introduces an electromagnetic sensor-based navigation system and develops a prototype for wrist arthroscopy, allowing real-time display of instrument positions on the arthroscopic view. This AR approach enhances surgical precision, comfort, and efficiency

Abbreviation: AR, augmented reality.

**Table 3:** RoB 2 quality assessment for randomized controlled trials.

Study	D1	D2	D3	D4	D5	Overall
Fang et al. [18]	Low	Some concerns	Low	Low	Low	Some concerns
Shim et al. [32]	Low	Some concerns	Low	Low	Low	Some concerns

**Table 4:** NIH quality assessment.

Study	1	2	3	4	5	6	7	8	9	Overall (out of 9)
Bautista et al. [15]	+	+		+	+	+		+	+	7
Chen/ et al. [10]	+		+		+	+		+		5
Dario et al. [17]	+	+	+	+	+	+		+	+	8
Fotouhi et al. [20]	+	+			+	+	+	+	+	7
Franzo et al. [33]	+			+	+	+		+		5
Garcia and Navarro [30]	+	+			+	+	+	+	+	7
Guo et al. [34]	+	+	+	+	+		+	+		7
Jeung et al. [16]	+			+	+	+		+	+	6
Korn et al. [31]	+	+	+	+	+	+		+		7
Moody et al. [22]	+			+	+	+	+	+		6
Rasool and Sourin [24]	+	+		+	+	+	+	+		7
Rose and Pedowitz [29]	+	+	+	+	+	+		+		7
Stetson et al. [25]	+	+	+	+	+	+		+	+	8
Tonet et al. [27]	+		+	+	+	+	+	+		7
Zemirline et al. [28]	+	+		+	+	+		+	+	7

## Data Availability

The data that support the findings of this study are available from the corresponding author upon reasonable request.

## Supporting Information

Supporting Table S1: Full search strategies. Detailed database-specific search strategies (PubMed, Embase, Scopus, and Web of Science) used to identify eligible studies for this review.
