# Supplementary material for: Advances in Augmented Reality in Sports Surgery: A Systematic Review
Source: Adv Orthop. 2025 Sep 10;2025:6707884. doi: 10.1155/aort/6707884 (PMC12443500; doi:10.1155/aort/6707884)
Supplement: Supporting Information — Additional supporting information can be found online in the Supporting Information section. [file 6707884.f1.docx]

**Supplementary Table S1. Full Search Strategies**

| Database | Search strategy |
| --- | --- |
| PubMed (MEDLINE) | ((((((((((((((((anterior cruciate ligament[Title/Abstract]) OR (menisc*[Title/Abstract])) OR (rotator cuff[Title/Abstract])) OR (biceps[Title/Abstract])) OR (triceps[Title/Abstract])) OR (clavic*[Title/Abstract])) OR (hip[Title/Abstract])) OR (foot[Title/Abstract])) OR (ankle[Title/Abstract])) OR (tendinopath*[Title/Abstract])) OR (achill*[Title/Abstract])) OR (shoulder[Title/Abstract])) OR (femoroacetabular[Title/Abstract])) OR (patella*[Title/Abstract])) OR (athlet*[Title/Abstract])) OR (osteochondrit*[Title/Abstract]) OR (sport*[Title/Abstract]) OR "arthroscop*"[tiab]) AND (((("augmented reality"[Title/Abstract]) OR ("extended reality"[Title/Abstract])) OR ("mixed reality"[Title/Abstract]))) |
| Embase | ((anterior cruciate ligament:ab,ti OR menisc*:ab,ti OR "rotator cuff":ab,ti OR biceps:ab,ti OR triceps:ab,ti OR clavic*:ab,ti OR hip:ab,ti OR foot:ab,ti OR ankle:ab,ti OR tendinopath*:ab,ti OR achill*:ab,ti OR shoulder:ab,ti OR femoroacetabular:ab,ti OR patella*:ab,ti OR athlet*:ab,ti OR osteochondrit*:ab,ti OR sport*:ab,ti OR arthroscop*:ab,ti) AND ("augmented reality":ab,ti OR "extended reality":ab,ti OR "mixed reality":ab,ti)) |
| Scopus | TITLE-ABS-KEY (anterior cruciate ligament OR menisc* OR "rotator cuff" OR biceps OR triceps OR clavic* OR hip OR foot OR ankle OR tendinopath* OR achill* OR shoulder OR femoroacetabular OR patella* OR athlet* OR osteochondrit* OR sport* OR arthroscop*) AND TITLE-ABS-KEY ("augmented reality" OR "extended reality" OR "mixed reality") |
| Web of Science | TS=(anterior cruciate ligament OR menisc* OR "rotator cuff" OR biceps OR triceps OR clavic* OR hip OR foot OR ankle OR tendinopath* OR achill* OR shoulder OR femoroacetabular OR patella* OR athlet* OR osteochondrit* OR sport* OR arthroscop*) AND TS=("augmented reality" OR "extended reality" OR "mixed reality") |

Searches were performed in January 2024 across PubMed (MEDLINE), Embase, Scopus, and Web of Science.
